# Complete Genome Sequence of a Novel Feline Astrovirus from a Domestic Cat in Hong Kong

**DOI:** 10.1128/genomeA.00708-13

**Published:** 2013-09-26

**Authors:** Susanna K. P. Lau, Patrick C. Y. Woo, Cyril C. Y. Yip, Ru Bai, Ying Wu, Herman Tse, Kwok-yung Yuen

**Affiliations:** Department of Microbiology, The University of Hong Kong, Hong Kong, Chinaa; State Key Laboratory of Emerging Infectious Diseases, The University of Hong Kong, Hong Kong, Chinab; Research Centre of Infection and Immunology, The University of Hong Kong, Hong Kong, Chinac; Carol Yu Centre of Infection, The University of Hong Kong, Hong Kong, Chinad

## Abstract

We report the first complete genome sequence of a feline astrovirus (FAstV), FAstV2 strain 1637F, identified from a domestic cat. The genome is 6,779 nucleotides (nt) in length and consists of three overlapping open reading frames (ORF1a-ORF1b-ORF2). Sequence analysis suggests that FAstV2 represents a new FAstV genotype that is closely related to human astroviruses.

## GENOME ANNOUNCEMENT

Astroviruses are small nonenveloped viruses with single-stranded positive-sense RNA genomes of 6.2 to 7.7 kb in length. The family *Astroviridae* contains two genera, *Mamastrovirus* and *Avastrovirus,* which infect mammals and birds, respectively. Astroviruses are important causes of diarrheal and occasionally extraintestinal disease in various animals, including humans (1–4). Because of their genetic diversity and recombination ability, astroviruses have the potential for zoonosis (5). Although feline astroviruses (FAstVs) have also been detected in the feces of cats with and without diarrhea (6–9), no complete genome sequence of a FAstV is available. In March 2012, we discovered a novel feline astrovirus, FAstV2 strain 1637F, which is closely related to human astroviruses (HAstVs), from a fecal sample from an asymptomatic domestic cat (*Felis catus*) in Hong Kong, and its complete genome was sequenced. This represents the first complete genome sequence of a FAstV.

The complete genome of FAstV2 1637F was amplified and sequenced using published strategies for single-stranded positive-sense RNA viruses (1, 10). RNA was extracted from the fecal sample with the EZ1 virus minikit (Qiagen, Germany) and used as a template. RNA was converted to cDNA by a combined random-priming and oligo(dT) priming strategy. The cDNA was amplified by degenerate primers designed by published strategies (10). The 5′ end of the viral genome was confirmed by rapid amplification of cDNA ends using the SMARTer RACE cDNA amplification kit (Clontech, USA). The sequences were assembled and manually edited to produce the final genome sequence.

The genome of FAstV2 strain 1637F is 6,779 nucleotides (nt) in length after excluding the polyadenylated tract, and the G+C content is 50 mol%. The genome organization is similar to those of other astrovirus genomes, with a 5′ untranslated region (5′ UTR) (29 nt), three overlapping open reading frames (ORFs), a 3′ UTR (81 nt), and a poly(A) tail. ORF1a (2,745 nt encoding 914 amino acids [aa]) encodes a nonstructural polyprotein with a conserved serine protease motif. ORF1b (1,548 nt encoding 515 aa) encodes an RNA-dependent RNA polymerase, which is expressed via a ribosomal frameshift at the ORF1a/1b junction, with a conserved “slippery heptamer” sequence, 5′-AAAAAAC-3′, near the 3′ end of ORF1a. ORF2 (2,445 nt) encodes a viral capsid precursor protein of 814 aa, in which a stretch of acidic residues is found near the C terminus. Multiple sequence alignment and phylogenetic analysis showed that the proteins encoded by the ORF1a and ORF1b of FAstV2 1637F are most closely related to those of HAstV genotype 6 (GenBank accession no. HM237363), with 53.4% and 78.6% aa identities, respectively, while the capsid precursor protein of FAstV2 1637F is most closely related to that of FAstV1 isolate Bristol (accession no. AF056197), with 70.8% aa identity; this supports the idea that FAstV2 represents a new FAstV genotype. While the complete genome and ORF1 sequences from FAstV1 are lacking, based on the complete genome of FAstV2, our analysis suggests that HAstVs have descended from a common ancestral animal astrovirus that is closely related to the FAstVs.

### Nucleotide sequence accession number.

The complete genome sequence of FAstV2 1637F has been submitted to GenBank under the accession no. KF499111.
